# Supplementary figures and images for: HSV-1 exploits host heterochromatin for nuclear egress
Source: J Cell Biol. 2023 Jul 26;222(9):e202304106. doi: 10.1083/jcb.202304106 (PMC10373338; doi:10.1083/jcb.202304106)

Source Data: Figure 1

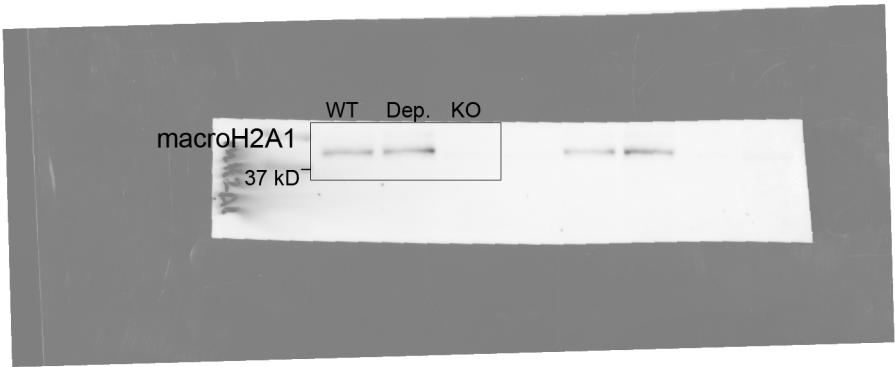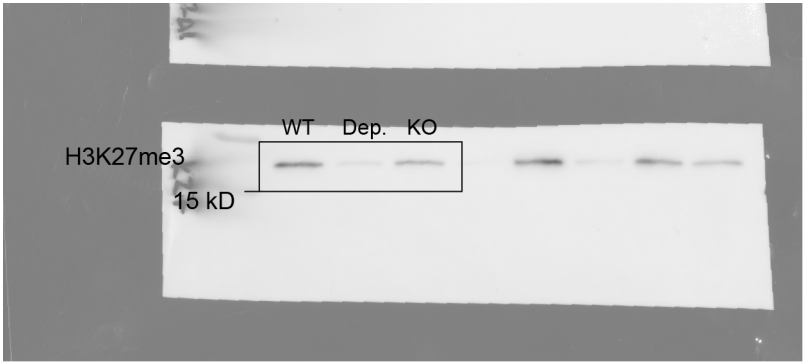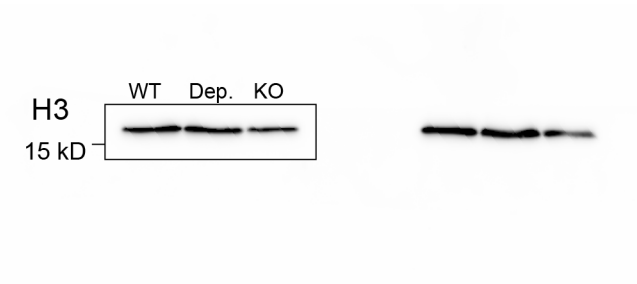

Supplement: SourceData F1 — is the source file for Fig. 1. [file JCB_202304106_SourceDataF1.pdf]

Source Data: Figure 3

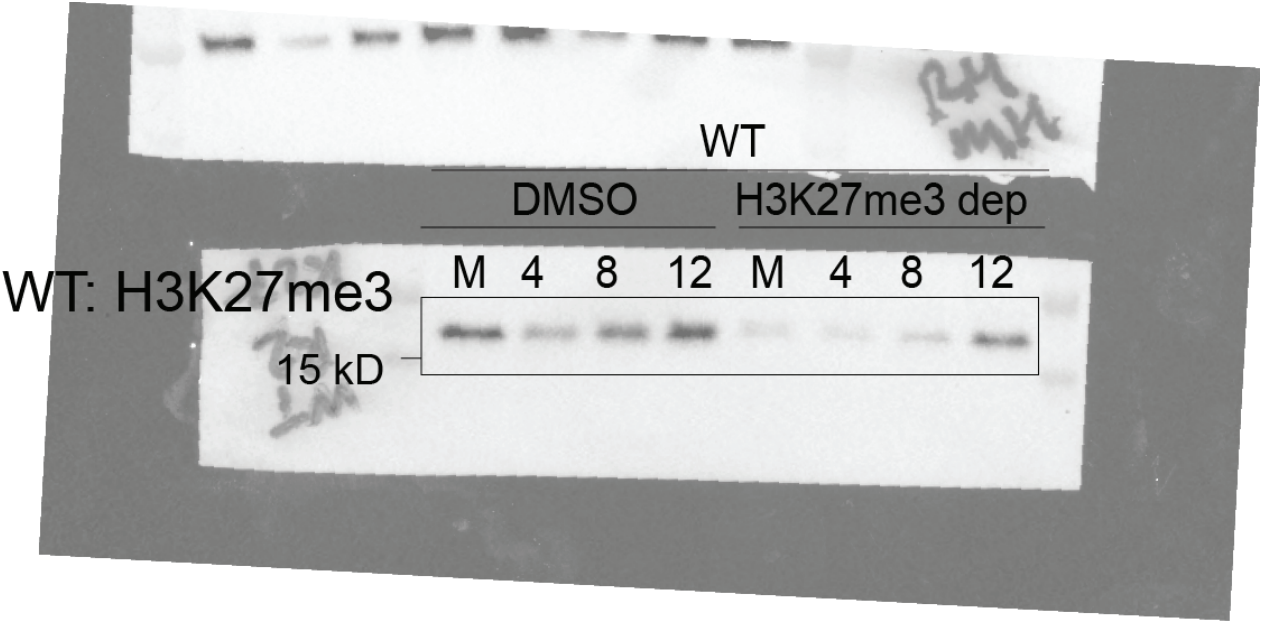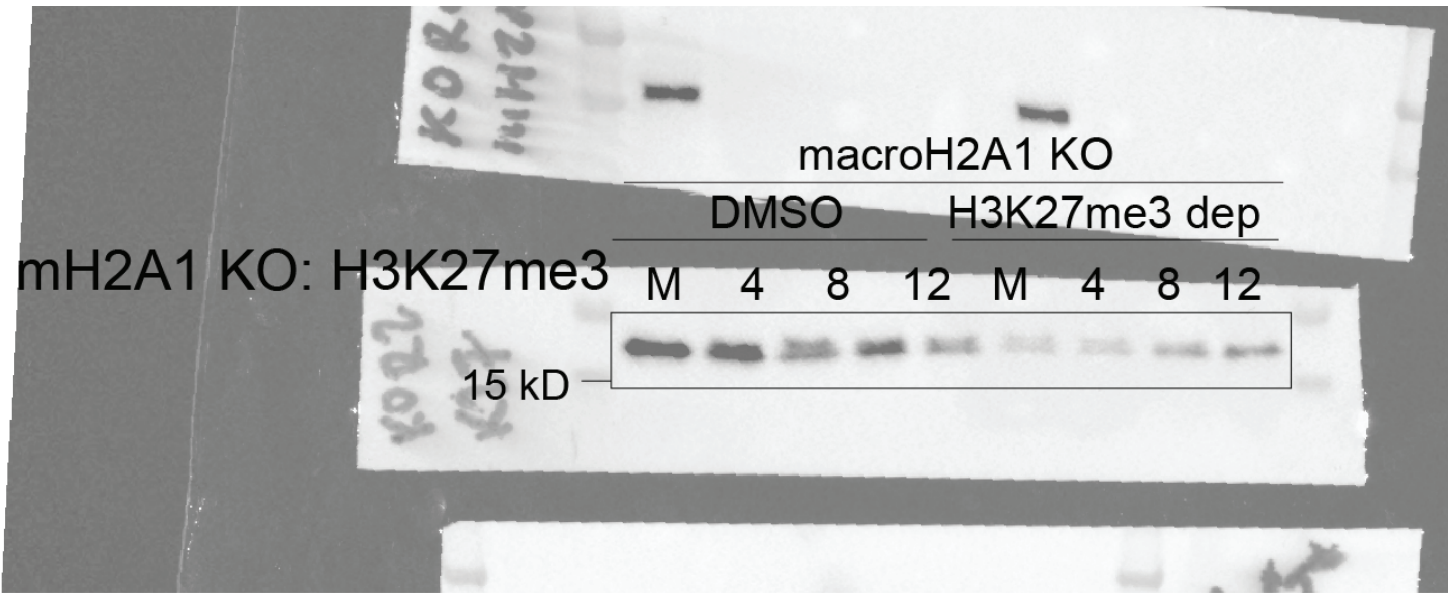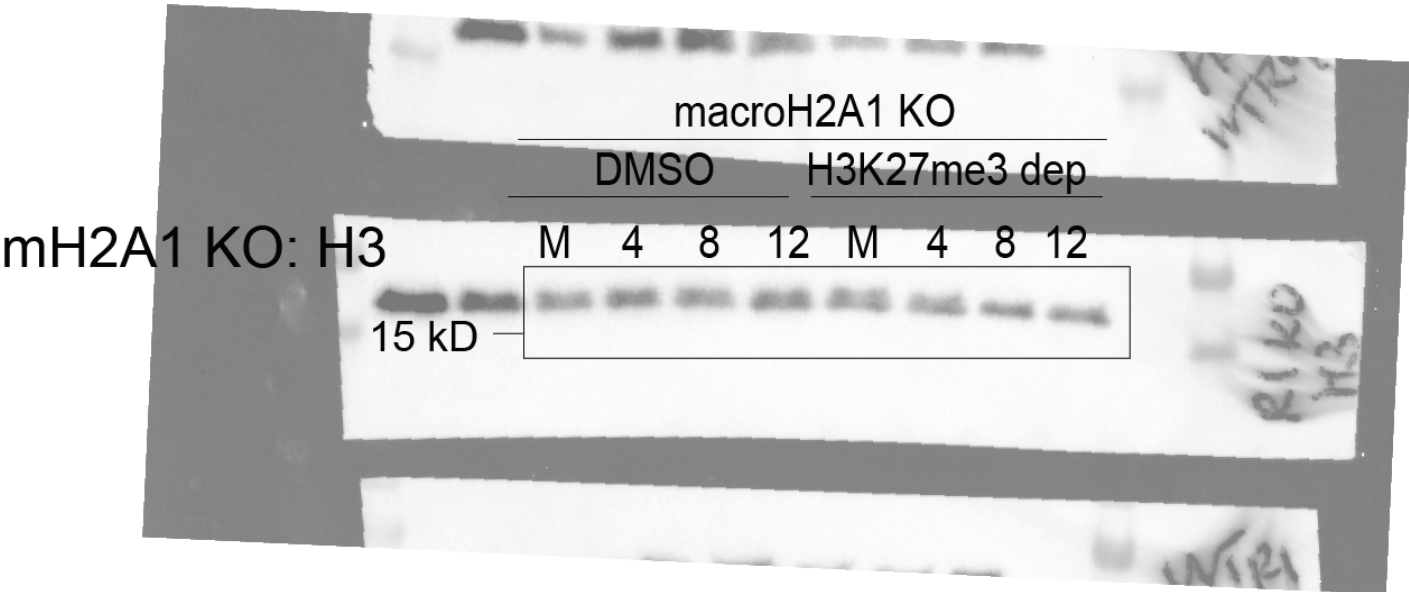

mH2A1 KO: mH2A1

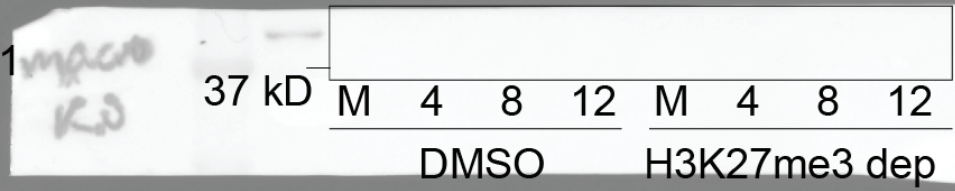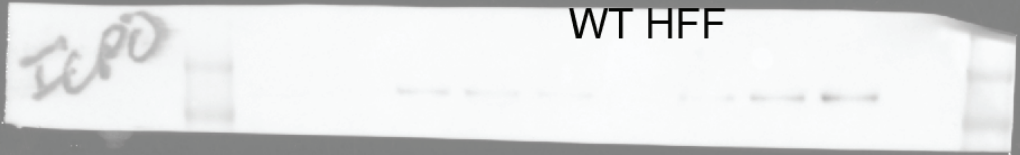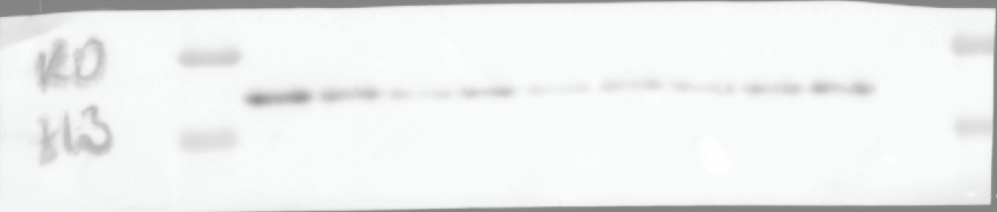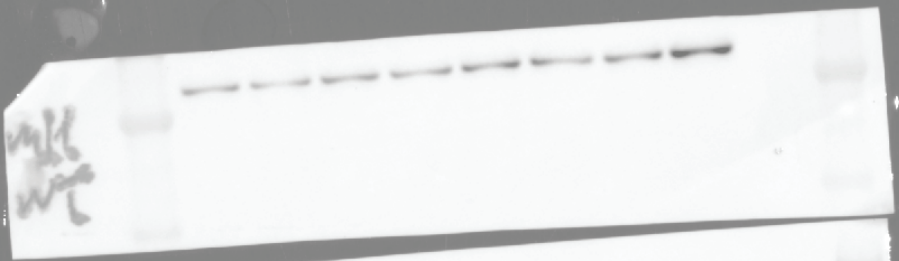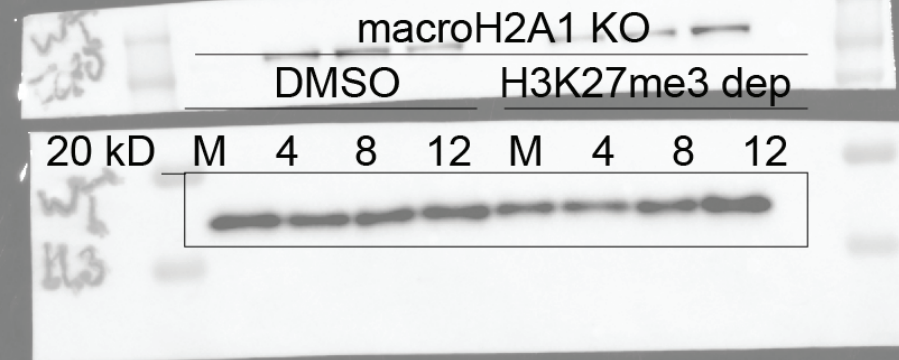

WT HFF: H3

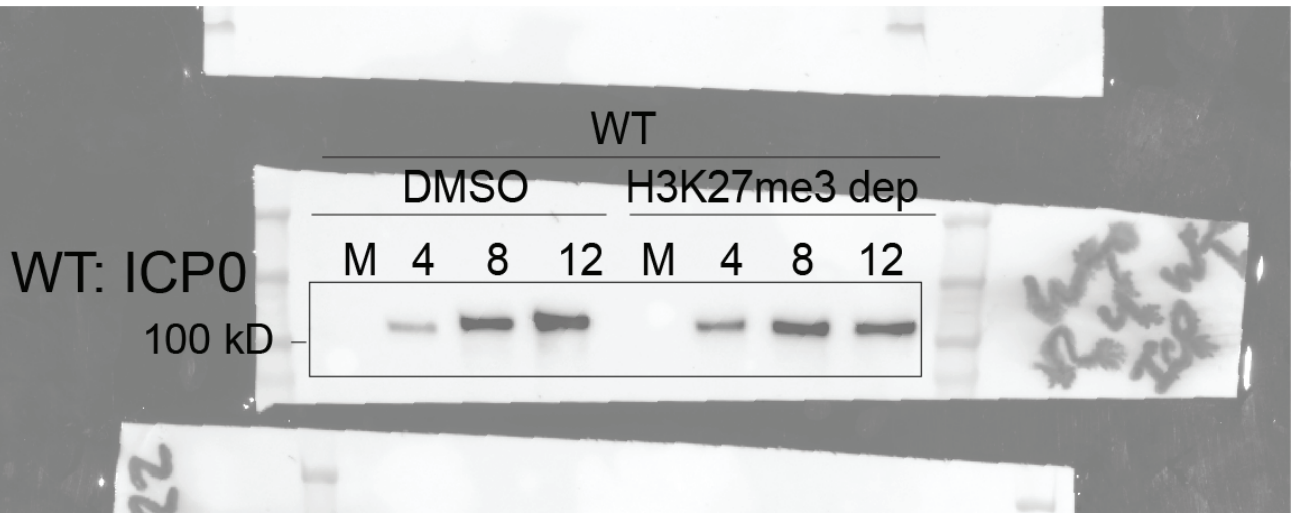

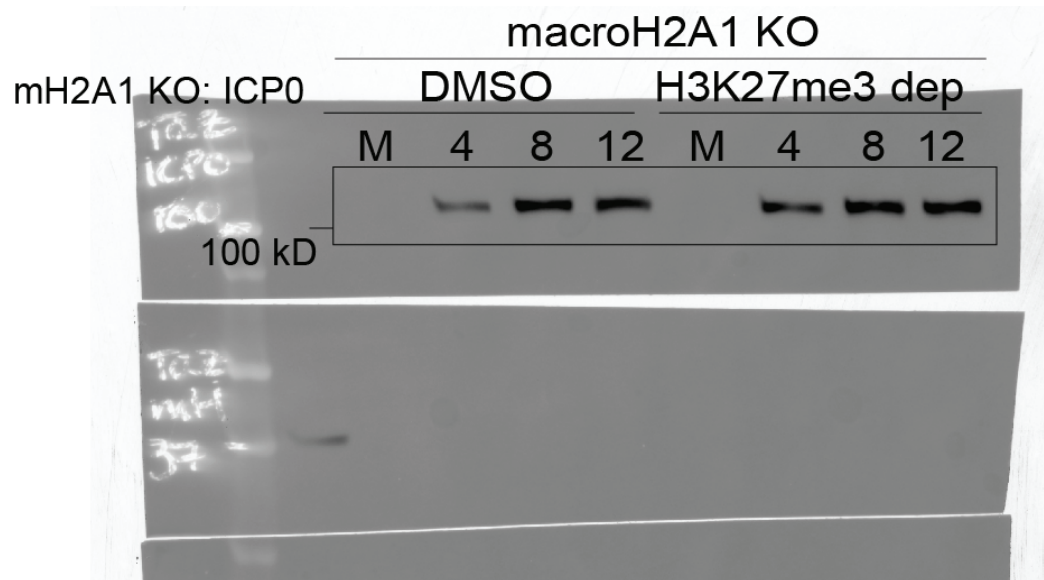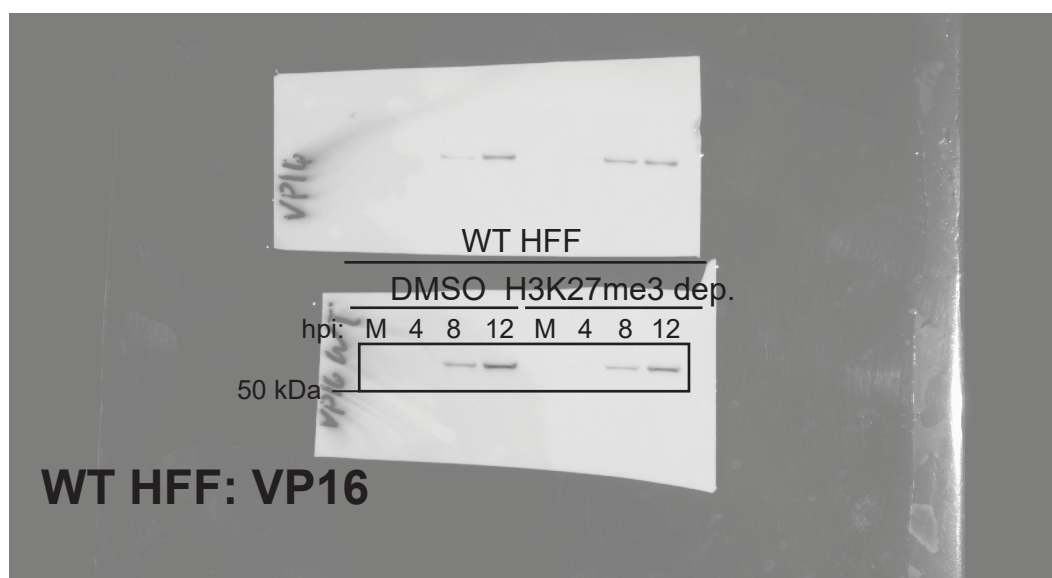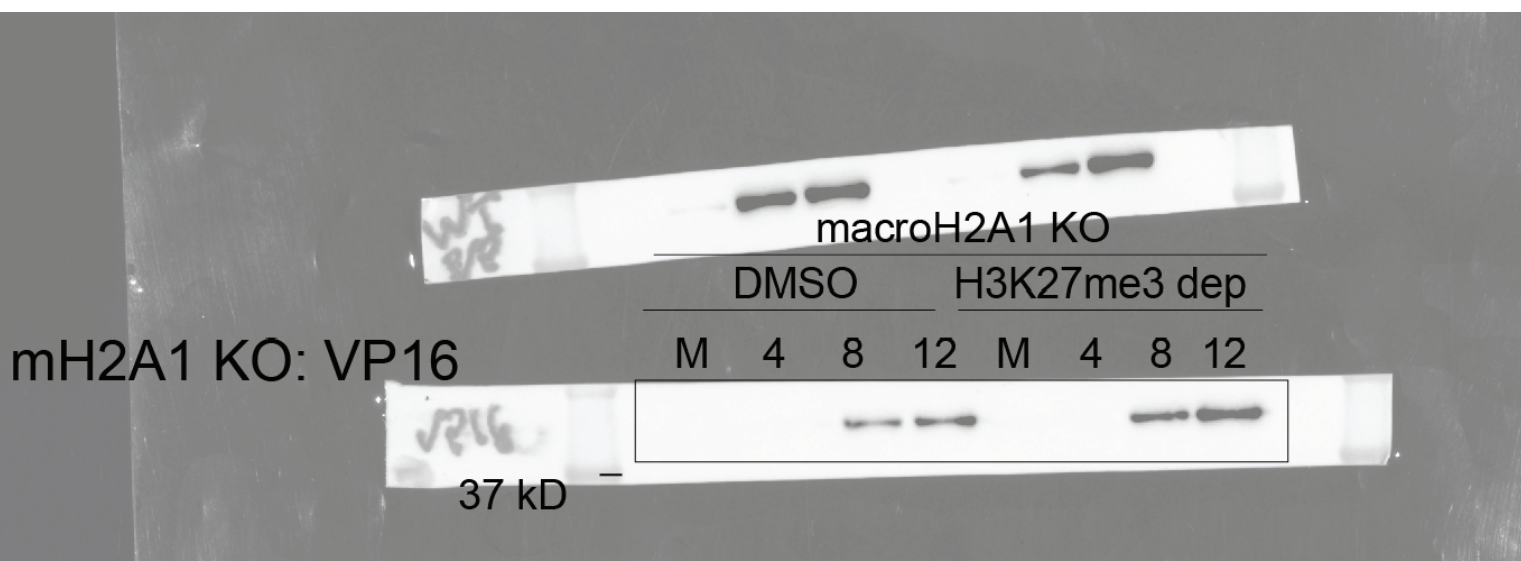

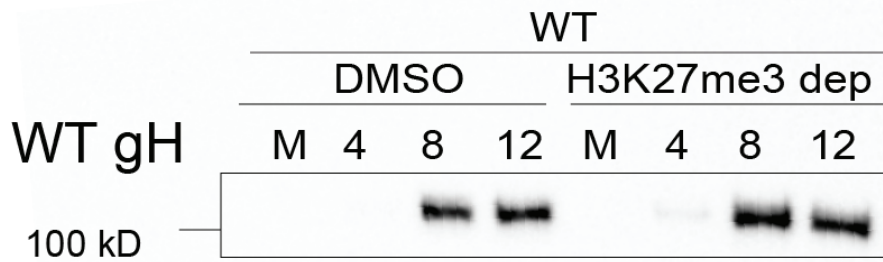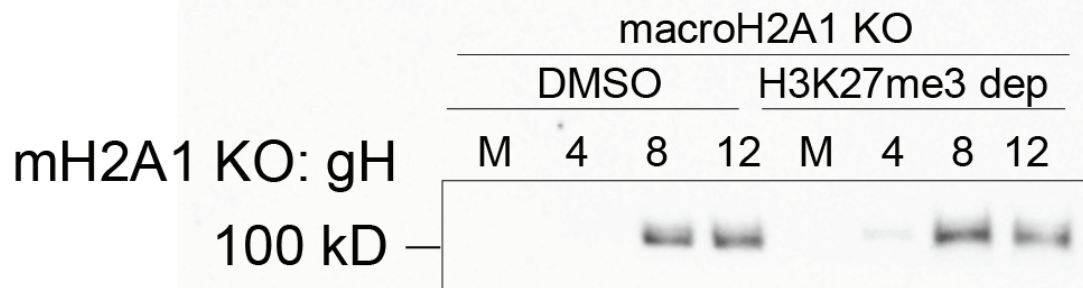

## WT HFF: mH2A1

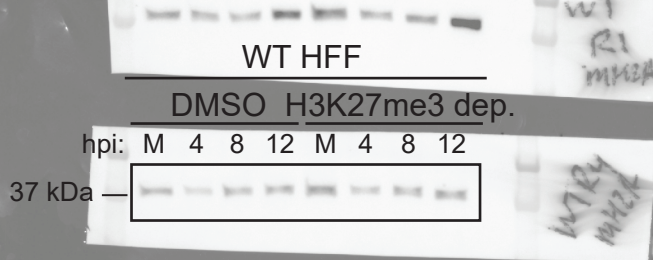

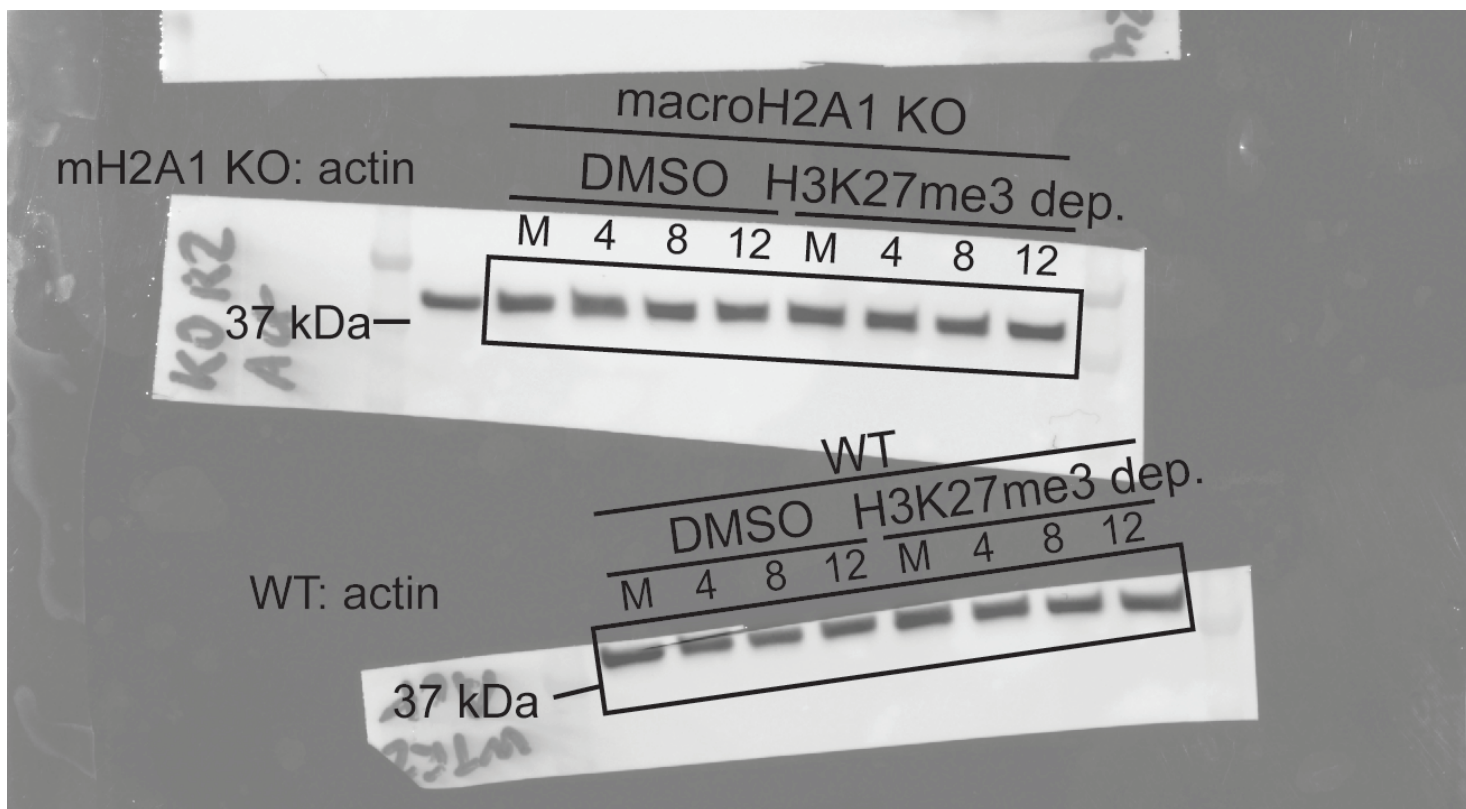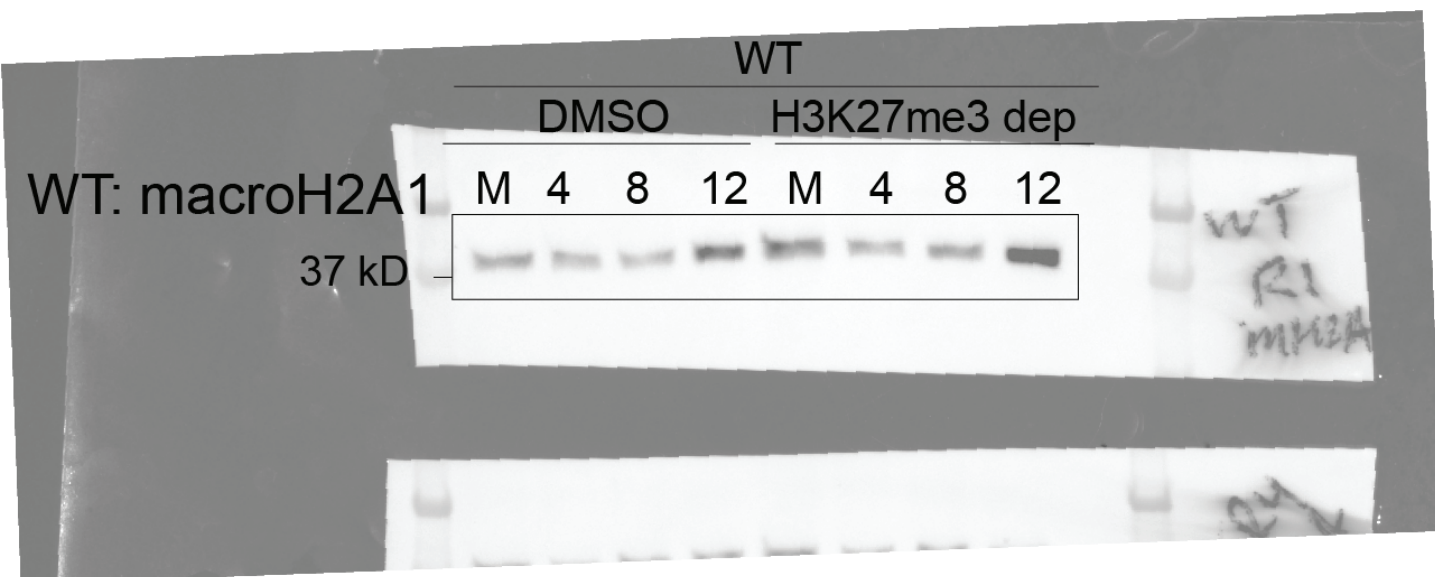

Supplement: SourceData F3 — is the source file for Fig. 3. [file JCB_202304106_SourceDataF3.pdf]

Source Data: Figure 4

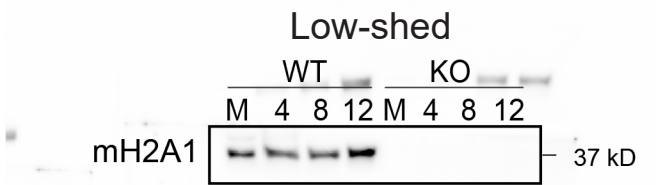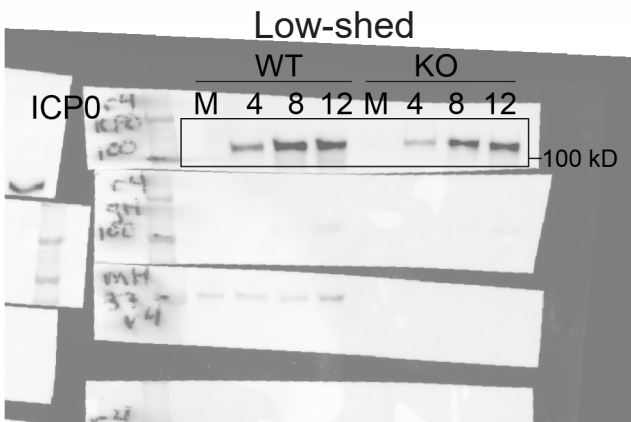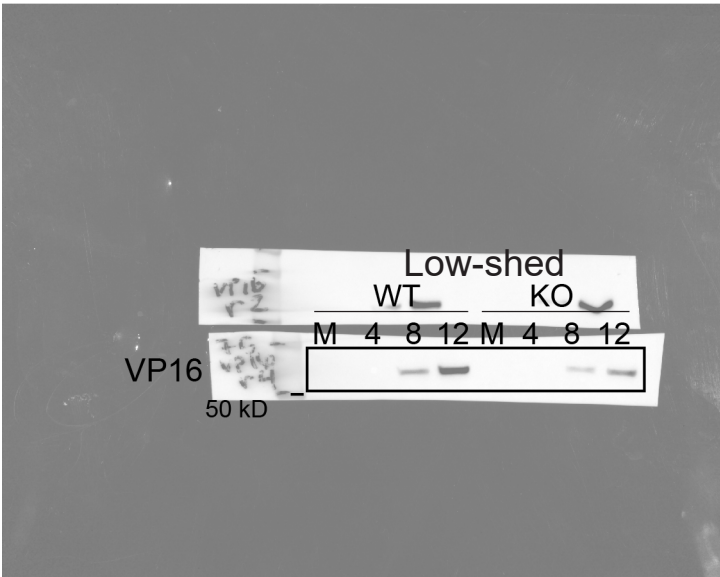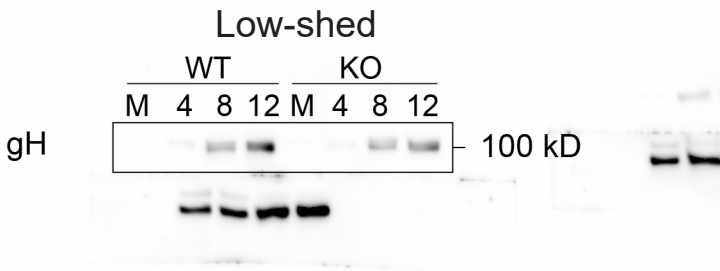

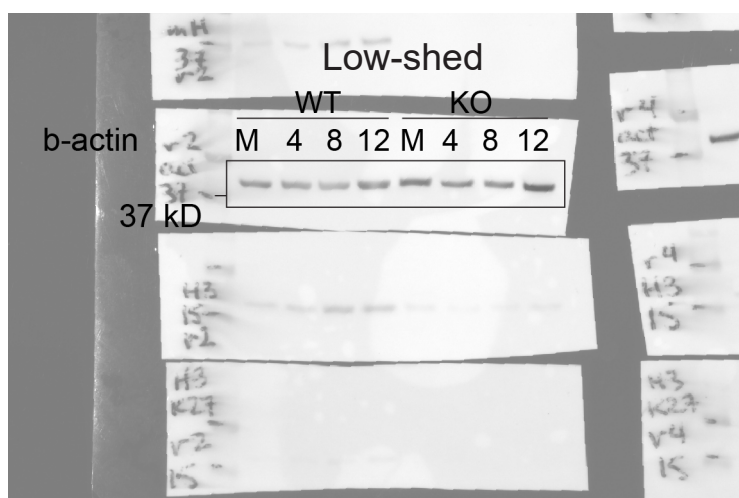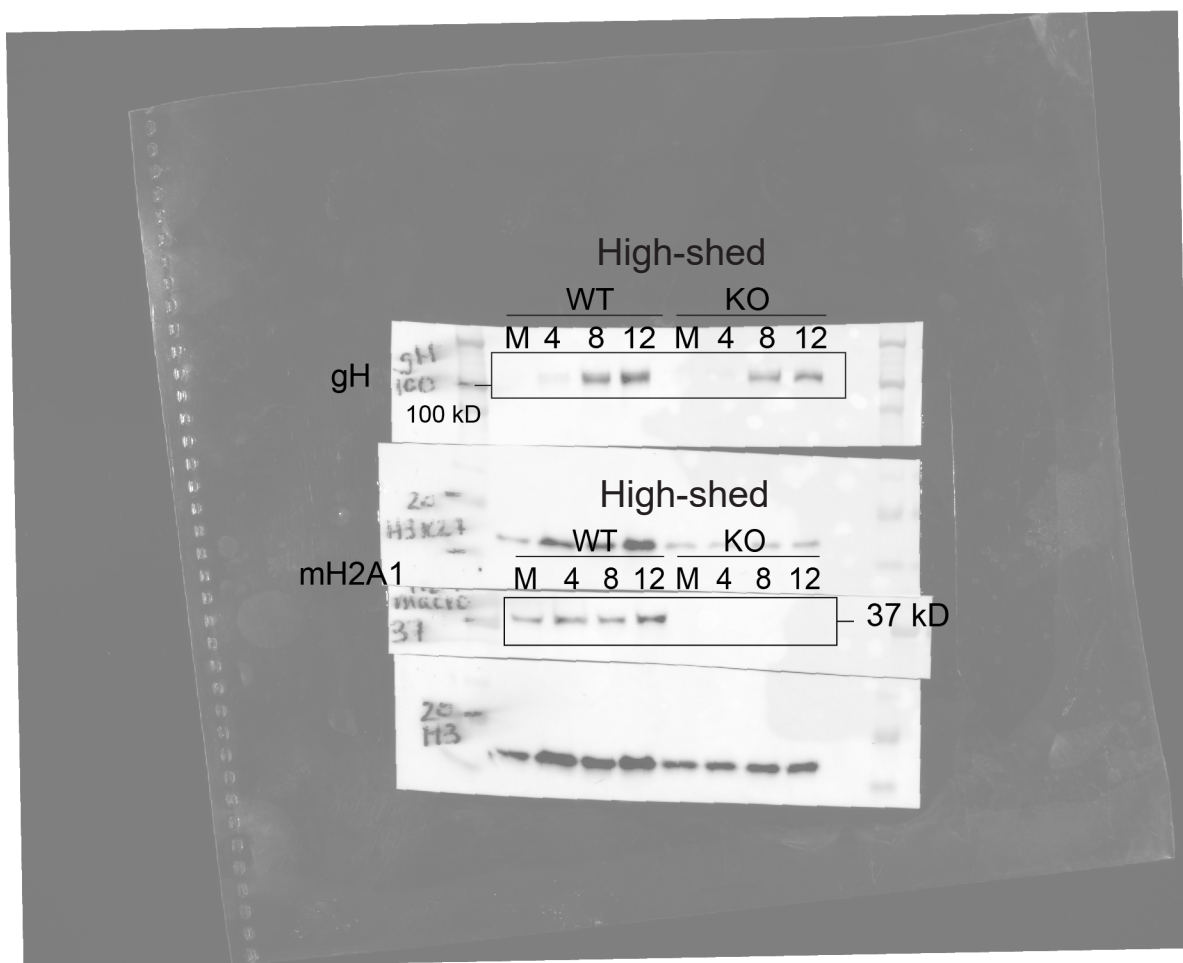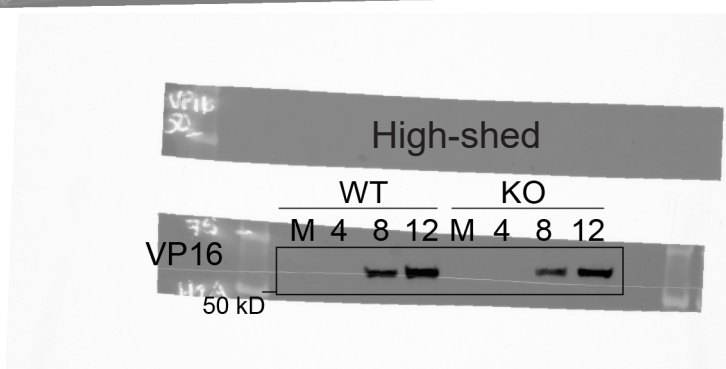

# High-shed

ICP0

| WT |   |   |    | KO |   |   |    |        |
|----|---|---|----|----|---|---|----|--------|
| M  | 4 | 8 | 12 | M  | 4 | 8 | 12 |        |
|    |   |   |    |    |   |   |    | 100 kD |

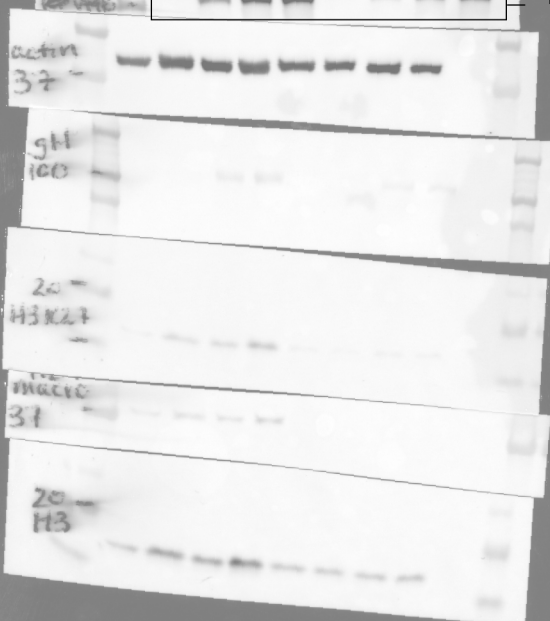

# High-shed

b-actin

| WT |   |   |    | KO |   |   |    |       |
|----|---|---|----|----|---|---|----|-------|
| M  | 4 | 8 | 12 | M  | 4 | 8 | 12 |       |
|    |   |   |    |    |   |   |    | 37 kD |

Supplement: SourceData F4 — is the source file for Fig. 4. [file JCB_202304106_SourceDataF4.pdf]

Source Data: Figure S1

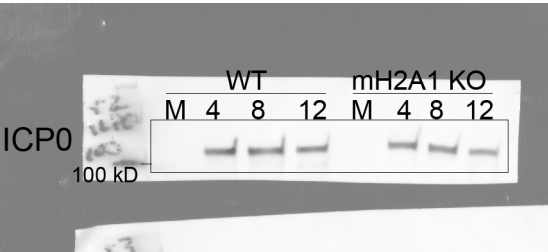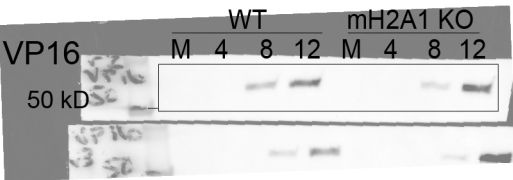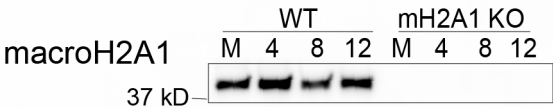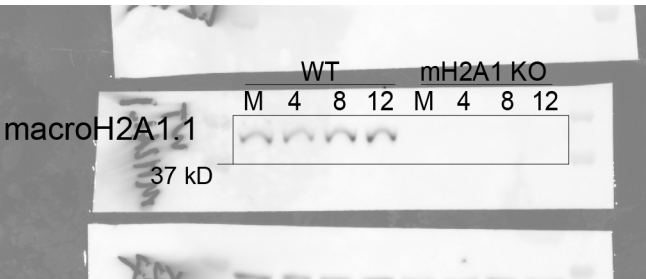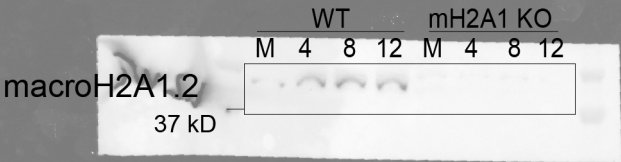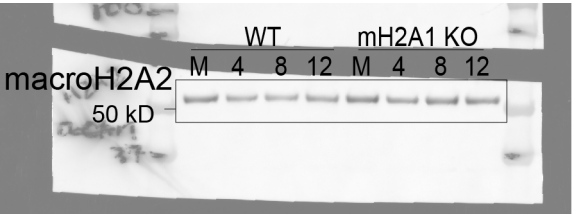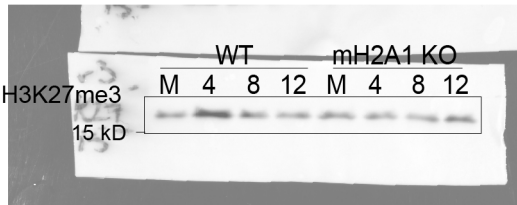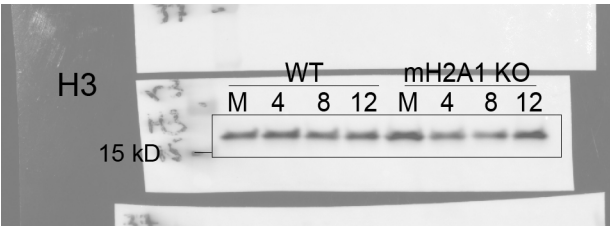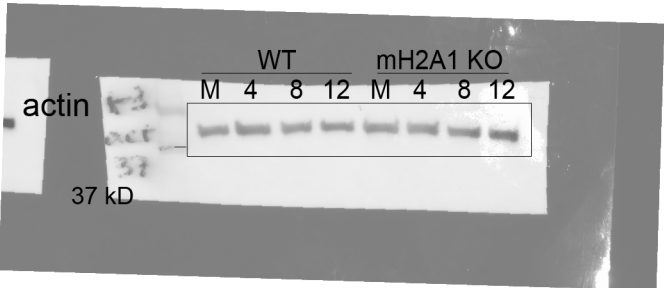

Supplement: SourceData FS1 — is the source file for Fig. S1. [file JCB_202304106_SourceDataFS1.pdf]

Source Data: Figure S4

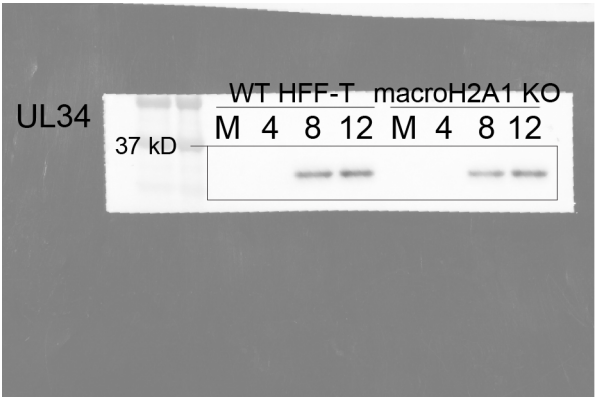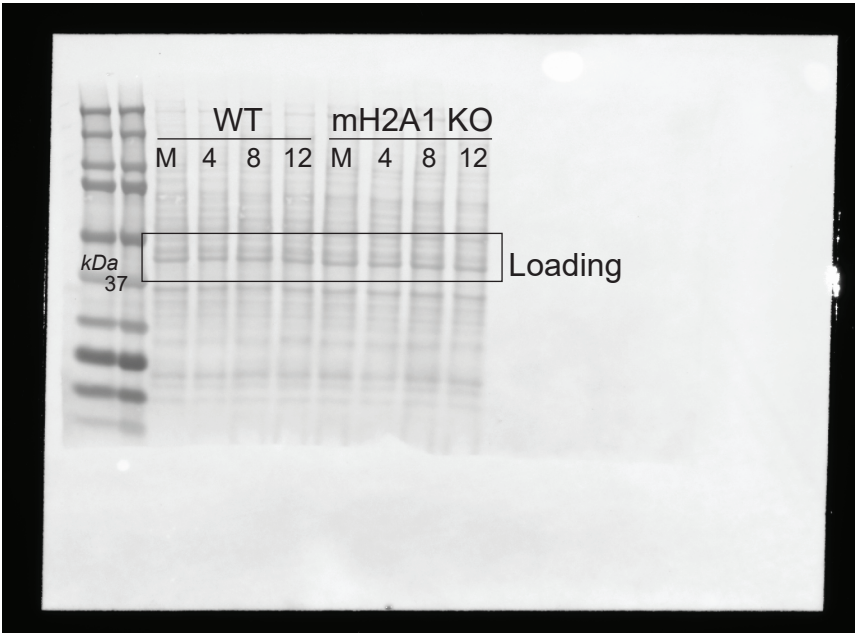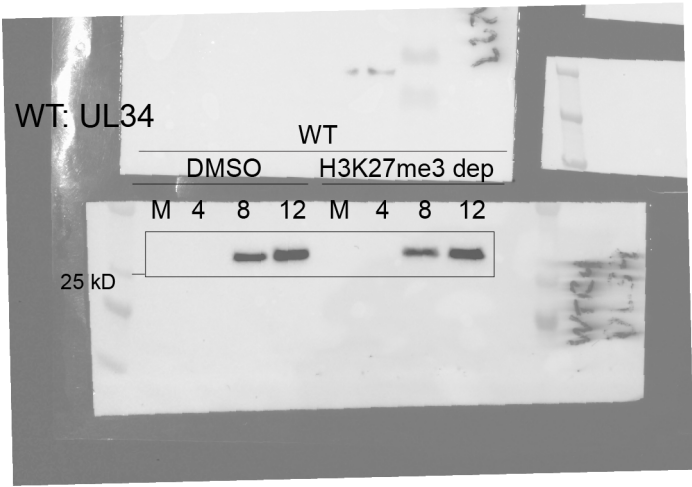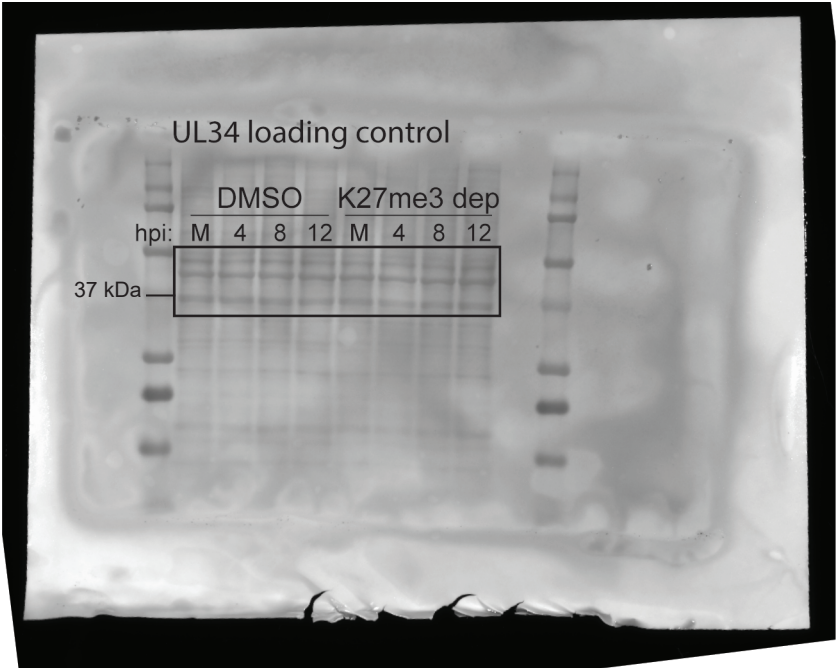

Supplement: SourceData FS4 — is the source file for Fig. S4. [file JCB_202304106_SourceDataFS4.pdf]

Source Data: Figure S5

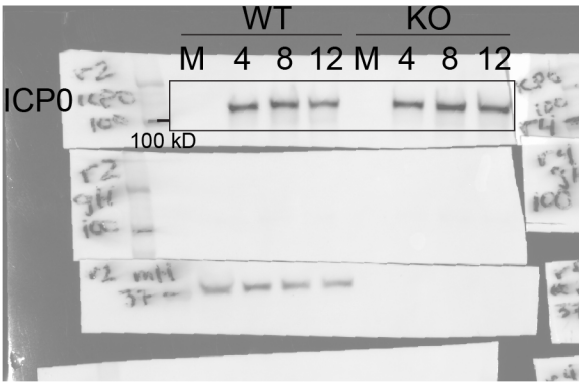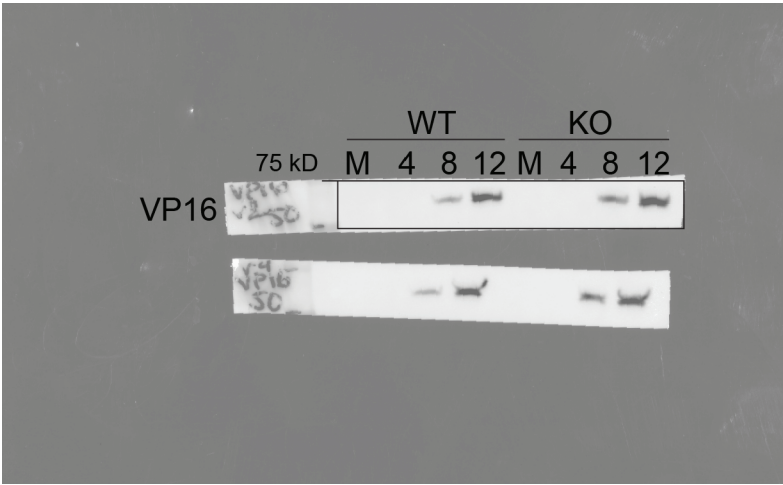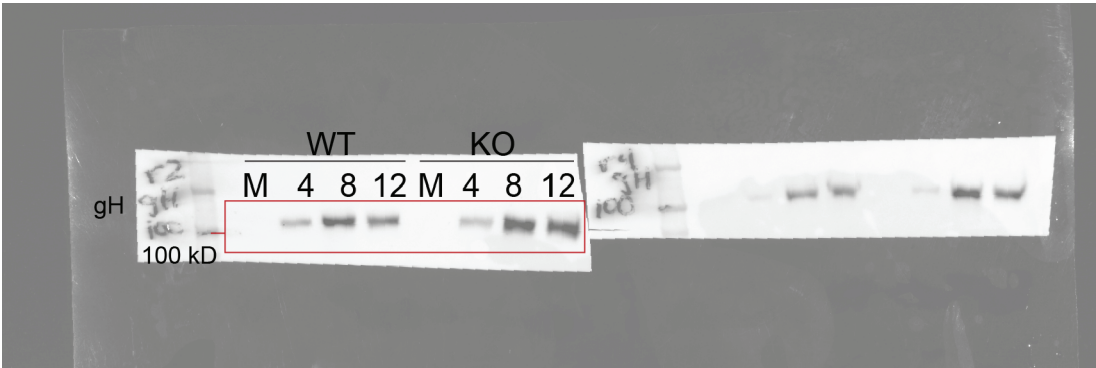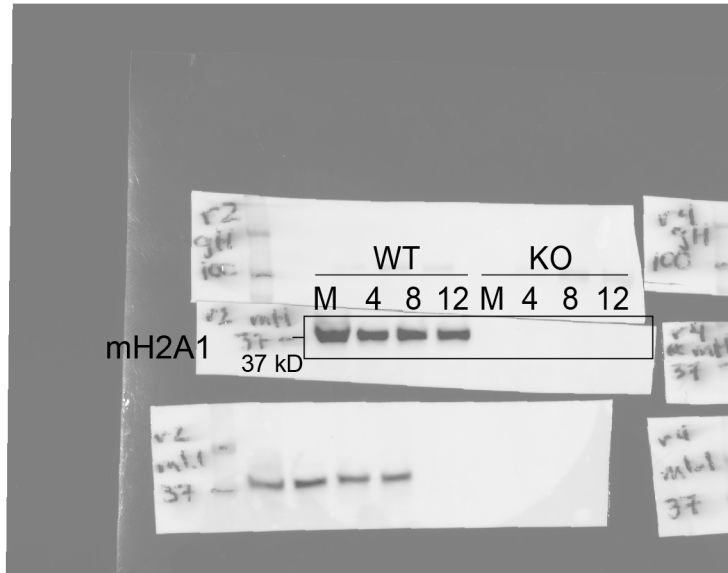

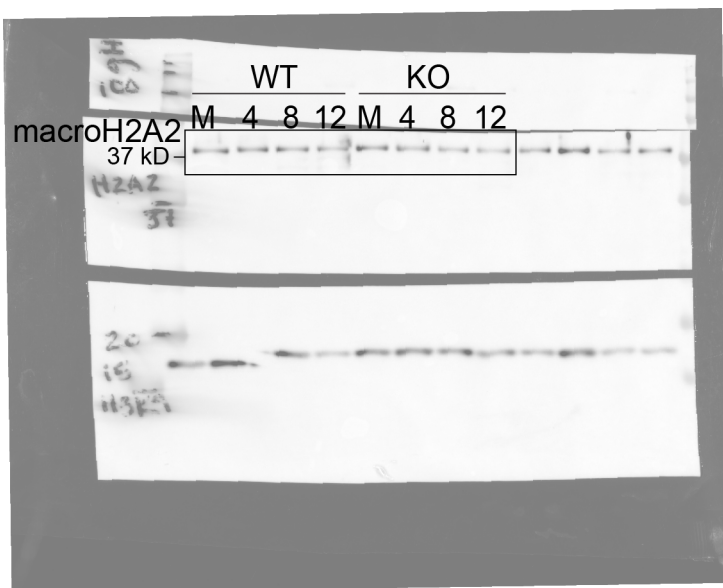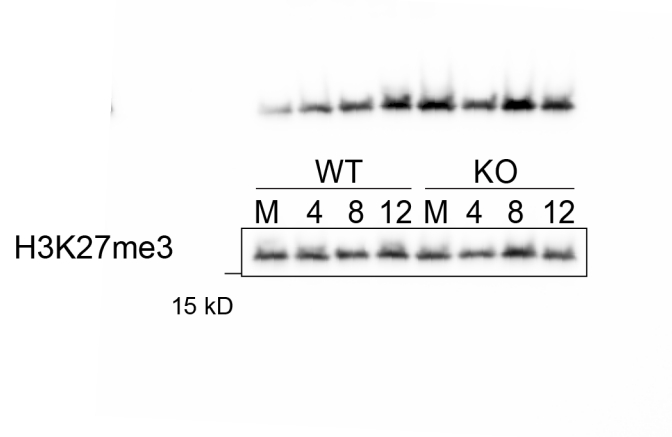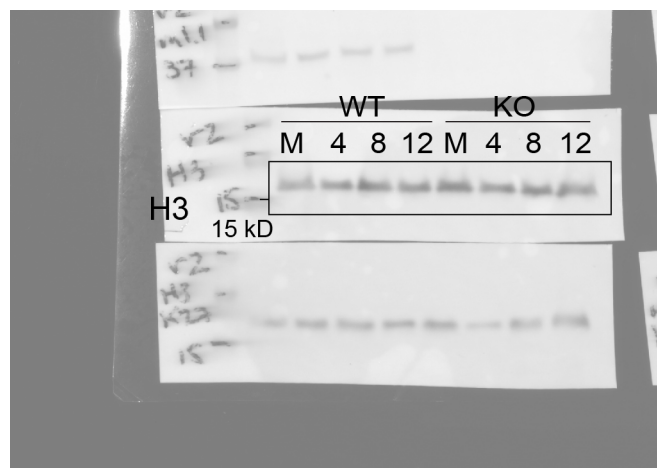

Supplement: SourceData FS5 — is the source file for Fig. S5. [file JCB_202304106_SourceDataFS5.pdf]
